# Delayed Signaling in Mitotic Checkpoints: Biological Mechanisms and Modeling Perspectives

**DOI:** 10.3390/biology15020122

**Published:** 2026-01-08

**Authors:** Bashar Ibrahim

**Affiliations:** 1 Centre for Applied Mathematics & Bioinformatics, Department of Mathematics & Natural Sciences, Gulf University for Science and Technology, Hawally 32093, Kuwait; ibrahim.b@gust.edu.kw; 2 Department of Mathematics and Computer Science, Friedrich Schiller University Jena, Fürstengraben, 07743 Jena, Germany; 3 European Virus Bioinformatics Center, Leutragraben 1, 07743 Jena, Germany

**Keywords:** mitotic checkpoints, time delays, spindle assembly checkpoint, spindle position checkpoint, delay differential equations

## Abstract

Time delays are intrinsic to mitotic regulation, particularly within the spindle assembly checkpoint (SAC) and the spindle position checkpoint (SPOC). These delays emerge from multi-step protein activation, molecular transport, force-dependent conformational transitions, and spatial redistribution of regulatory complexes. They span seconds to minutes and strongly influence checkpoint activation, maintenance, and silencing. Increasing evidence shows that such delayed processes shape mitotic timing, checkpoint robustness, and cell-fate decisions. While classical ordinary differential equation (ODE) models assume instantaneous biochemical responses, delay differential equations (DDEs) provide a natural framework for representing these finite timescales by explicitly incorporating system history. Recent DDE-based studies have revealed how delayed signaling contributes to bistability, oscillatory responses, prolonged mitotic arrest, and variability in checkpoint outputs. This review summarizes the biological origins of delays in SAC and SPOC, including Mad2 activation, MCC assembly and turnover, APC/C reactivation, tension maturation at kinetochores, and Bfa1–Bub2 regulation of Tem1. The article further discusses how mechanistic models with explicit delays improve our understanding of SAC–SPOC ordering, error-correction dynamics, and mitotic exit control. Finally, open challenges and future directions are outlined for integrative delay-aware modeling that unifies biochemical, mechanical, and spatial processes to better explain checkpoint function and chromosomal stability.

## Simple Summary

When cells divide, they must carefully separate their genetic material to avoid errors that can lead to diseases such as cancer. This process is monitored by internal quality-control systems called checkpoints, which act like safety inspectors, ensuring that chromosomes are properly attached, under tension, and correctly positioned before division is allowed to proceed. These checkpoints do not work instantaneously. Instead, there are natural time delays as molecular signals are generated, transported within the cell, and gradually interpreted. Traditional mathematical models of cell division often assume that these processes happen immediately, which overlooks important aspects of real cellular behavior. This review examines the biological sources of time delays in checkpoint control, including the time required for proteins to change shape, move to different locations inside the cell, and accumulate to effective levels. By using mathematical models that explicitly include these delays, researchers can better understand why some cells divide more slowly than others, why checkpoint signals can persist or fluctuate over time, and why errors sometimes escape detection. This improved understanding helps explain how faulty timing contributes to chromosomal instability in cancer cells and may support future strategies for preventing or controlling abnormal cell division.

## 1. Introduction

Accurate chromosome segregation during mitosis is essential for maintaining genomic stability. The cell employs sophisticated surveillance modules such as the spindle assembly checkpoint (SAC) and the spindle position checkpoint (SPOC) to prevent premature anaphase onset or exit from mitosis [1,2,3]. These checkpoints rely on multiscale signaling events, including protein recruitment at kinetochores, assembly of the mitotic checkpoint complex (MCC), conformational changes under tension, spatial transport, and regulated degradation of cell-cycle regulators [4,5,6,7,8,9,10,11]. Many of these processes unfold over finite timescales: phosphorylation cycles and conformational switching typically occur on the order of seconds, whereas multi-step complex assembly, long-range molecular transport, and mitotic exit can require tens of seconds to several minutes. These intrinsic delays imply that checkpoint decisions depend on the recent history of attachment, tension, and spindle orientation, rather than on instantaneous snapshots alone [1,3,10,12,13].

Both SAC and SPOC operate across molecular, subcellular, and cellular levels of organization. At unattached or low-tension kinetochores, checkpoint proteins such as Mad1, Mad2, Bub1, BubR1, and Bub3 are recruited and assembled into higher-order complexes that catalyze formation of the MCC, which sequesters Cdc20 and inhibits the anaphase-promoting complex/cyclosome (APC/C) [4,5,6,14,15,16]. These processes involve template-based conformational conversion of Mad2 [4,5], catalyst-driven MCC assembly and turnover [6,15,16], spatial transport between kinetochores, spindle, and cytoplasm [1,10,11], and controlled degradation of cyclin and securin by APC/C once the checkpoint is satisfied [17,18]. SPOC signaling, in turn, senses spindle mispositioning and integrates cortical and spindle pole signals to regulate Tem1 and the mitotic exit network, thereby coordinating spindle orientation with cytokinesis [3]. These intertwined processes introduce nontrivial time lags at multiple stages of checkpoint activation and silencing.

Classical checkpoint models are predominantly formulated as systems of ordinary differential equations (ODEs) [6,9,17,19,20,21,22]. In such models, biochemical reactions are assumed to occur effectively instantaneously given the current state of the system, or at least on timescales that are negligible relative to the modeled dynamics. ODE-based SAC models have been highly successful in clarifying core feedback structures, identifying bistable switching mechanisms, and predicting the influence of key regulators such as Aurora B, APC/C, and MCC [9,17,19,22,23]. However, mounting experimental evidence reveals substantial intracellular delays in SAC activation, MCC accumulation and turnover, APC/C inhibition and reactivation, and SPOC signaling. These include delays associated with multi-step protein assembly, template-based conformational changes, processive phosphorylation and dephosphorylation, microtubule attachment dynamics, mechanical tension buildup, and spatial redistribution of regulators across micrometer-scale cellular compartments [1,3,7,10,12,13,24,25].

Such delays can profoundly alter checkpoint dynamics. Even in simple regulatory motifs, time lags are known to generate oscillations, bistability, long transients, resonance phenomena, and chaotic behavior [24,25,26,27]. In the context of mitotic checkpoints, delays can influence how rapidly cells respond to attachment errors, how robustly they maintain arrest in the presence of persistent insults, and how reliably they transition out of arrest once all conditions are satisfied. They may also underlie observed cell-to-cell variability in mitotic timing, the emergence of prolonged or arrested mitoses, and the appearance of complex temporal patterns in checkpoint signaling [3,9,22,28]. Capturing these effects within a purely ODE-based framework typically requires ad hoc chains of intermediate variables or large numbers of effective steps, which can obscure mechanistic interpretation and complicate parameter estimation.

Delay differential equations (DDEs) provide a natural mathematical framework for explicitly incorporating time lags in regulatory networks [25,26,27]. In a DDE, the rate of change in a state variable depends not only on its current value but also on its history over one or more delay intervals. This formalism allows direct representation of transport times, multi-step assembly processes, delayed feedback, and memory effects without resorting to excessively high-dimensional ODE approximations. Over the past decades, DDEs have been applied to a wide variety of physiological and cellular systems, including hematopoiesis, neural dynamics, circadian rhythms, and gene regulatory circuits [24,25]. For mitotic checkpoints, DDE-based modeling is particularly appealing because it aligns closely with the biological reality of spatially distributed, multi-step, and mechanically coupled processes that do not respond instantaneously to perturbations [3,9,22,28].

Despite these advantages, the systematic use of DDEs in SAC and SPOC modeling remains relatively limited compared with ODE-based approaches. Existing checkpoint models with delays often introduce a single effective time lag or focus on specific regulatory modules, while comprehensive, multi-delay formulations that integrate SAC, SPOC, and mechanochemical couplings are still emerging [3,12,13,22,28]. Moreover, DDE models raise their own challenges, including increased mathematical complexity, nonlocal phase space, subtleties in numerical simulation, and difficulties in parameter identifiability when delays are not directly measurable [25,26,27]. At the same time, advances in bifurcation theory, numerical continuation for DDEs, and hybrid AI–mechanistic modeling open new opportunities for analyzing delay-induced dynamics and for inferring effective delays from experimental data [17,26,27,29,30,31].

**Figure 1:**
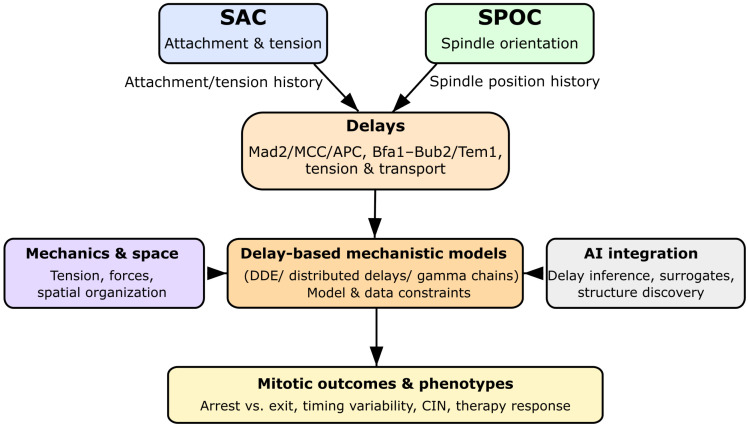
Graphical summary of the review. SAC and SPOC integrate attachment, tension, and spindle orientation over finite timescales. These biological delays are explicitly represented in delay differential equation (DDE) models, which can be coupled to mechanochemical descriptions and AI-based inference frameworks. This delay-aware perspective connects molecular mechanisms to mitotic outcomes, chromosomal instability, and therapy response.

The present article surveys and contextualizes DDE-based approaches to mitotic checkpoint modeling, with a particular focus on SAC and SPOC. First, the biological sources of delay are discussed, emphasizing how molecular transport, conformational changes, multi-step assembly, and mechanical cues generate non-negligible time lags in checkpoint signaling. The article then introduces mathematical formulations of DDEs relevant to checkpoint networks, including discrete and distributed delays, gamma-chain approximations, and hybrid ODE–DDE architectures [24,25,26,27]. Subsequently, existing DDE-based SAC and SPOC models are reviewed and compared, highlighting how delays reshape key dynamical features such as bistability, oscillations, and chaotic regimes [3,9,13,22,28,32]. Finally, open challenges and future directions are outlined, including the integration of mechanochemical and spatial effects, the combination of DDEs with data-driven and AI-assisted approaches, and the construction of unified delay-aware frameworks that capture the coupled dynamics of SAC and SPOC in realistic cellular contexts [3,17,30,31]. An overview is shown in Figure 1. As a review article, this manuscript emphasizes representative and illustrative numerical examples that highlight generic dynamical mechanisms, rather than reproducing exhaustive simulations for every existing checkpoint model. Key delay-related terminology used throughout this review is summarized in Box 1.

Box 1 **Glossary of Key Terms in Delay-Based Checkpoint Modeling.**

**Fixed Delay (τ):** A single, discrete time lag where the rate of change at time *t* depends on the state at t−τ.

**Distributed Delay:** A weighted memory of past states, represented by an integral kernel K(s) capturing a distribution of lag times.

**Gamma-Chain Approximation:** A sequence of first-order ODE compartments approximating a distributed delay with tunable variance.

**Mechanochemical Delay:** A finite timescale associated with force buildup, tension sensing, and mechanotransduction at kinetochores.

**Checkpoint Memory:** Persistence of SAC/SPOC signaling after the underlying cue has disappeared, caused by delayed MCC/APC or Tem1/Bfa1 dynamics.

**History Function:** Initial condition of a DDE defined over $\left[t-\tau_{\max},t\right]$, representing the system’s past trajectory.

**Hopf Bifurcation:** Delay-induced transition from a stable steady state to oscillations.

**Chaos in DDEs:** Aperiodic, sensitive dynamics arising from large delays and strong nonlinear feedback.

## 2. Biological Origins of Time Delays in Mitotic Checkpoints

Time delays in mitotic checkpoints arise from molecular, spatial, and mechanochemical processes that operate on finite, biologically meaningful timescales. These temporal features shape how cells detect spindle defects, sustain arrest, and commit to anaphase or mitotic exit [3,10,33,34]. Below, the main experimentally supported mechanisms that generate such delays in SAC and SPOC function are summarized.

**Figure 2:**
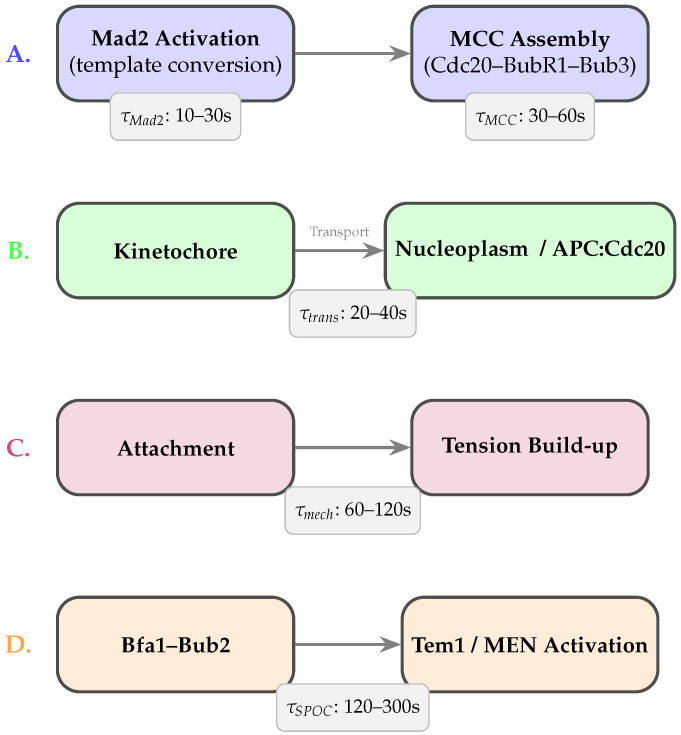
Biological sources of time delays in SAC and SPOC. (**A**) Molecular delays arise from multi-step Mad2 activation and MCC assembly. (**B**) Spatial delays reflect finite transport times required for MCC to reach APC/C. (**C**) Mechanochemical delays originate from tension buildup and force-dependent error-correction pathways. (**D**) SPOC-specific delays arise from Bfa1–Bub2 regulation of Tem1 and the MEN. Approximate timescales are shown for each process.

As illustrated in Figure 2, four major classes of processes contribute to checkpoint delays: (A) multi-step molecular conversions, (B) spatial transport across subcellular compartments, (C) mechanochemical tension maturation, and  (D) SPOC-specific regulatory dynamics.

### 2.1. Molecular Conversion and Assembly Delays

SAC activation begins at unattached or low-tension kinetochores with the catalytic conversion of open Mad2 (O-Mad2) to closed Mad2 (C-Mad2) by the Mad1–Mad2 complex [4,5]. This conformational conversion, followed by sequential MCC assembly with Cdc20, BubR1, and Bub3 [6,10,14], introduces characteristic latencies on the order of tens to hundreds of seconds. MCC-mediated APC/C inhibition and APC15-dependent turnover further extend this timescale [15,16]. Together, these processes create a temporal gap between the detection of attachment defects and full APC/C inhibition [19,22]. Aurora B-driven phosphorylation cycles add additional delays, as repeated phosphorylation–dephosphorylation events integrate mechanical cues at kinetochores [8,9,10].

### 2.2. Spatial Transport and Compartmental Delays

SAC signaling is spatially distributed across kinetochores, spindle structures, and the cytoplasm [1,7]. Checkpoint components must be transported away from kinetochores—via dynein-mediated motion, diffusion, or active trafficking—before inhibiting nucleoplasmic APC/C [11]. Transport distances and subcellular geometry impose characteristic delays, generating communication lags between kinetochore-level states and global APC/C activity [10].

### 2.3. Mechanochemical Delays at Kinetochores

Stabilizing kinetochore–microtubule attachments requires gradual tension buildup driven by microtubule dynamics, motor activity, and the viscoelastic behavior of chromatin and outer-kinetochore complexes [7,10]. Phosphoregulation of Ndc80, KNL1, and associated proteins integrates these mechanical cues over finite timescales. Even under correct attachments, force-dependent conformational changes and delayed phosphatase action can postpone SAC silencing [3,13]. These mechanochemical latencies naturally correspond to explicit delays in coarse-grained checkpoint models and are strongly linked to chromosomal instability when perturbed.

### 2.4. Multi-Step Signaling Cascade Delays

SAC signaling involves cascades of recruitment, phosphorylation, and binding events among Bub1, BubR1, Bub3, and Cdc20 [5,6]. These multi-step interactions act as temporal filters that smooth short-lived fluctuations but also introduce characteristic lag times due to sequential phosphorylation and competitive interactions at APC/C [14]. While classical ODEs approximate these cascades using chains of intermediates, explicit delays or distributed-delay kernels more naturally capture their coarse-grained timing [22,24,25].

### 2.5. Checkpoint Crosstalk and SPOC-Specific Delays

The SPOC introduces additional delays associated with spindle orientation sensing. The Bfa1–Bub2 complex regulates Tem1 and the mitotic exit network (MEN) through phosphorylation and relocalization events at spindle pole bodies [3]. These processes operate on minute-scale timescales and therefore differ from SAC-associated delays. Crosstalk between SAC and SPOC thus involves asymmetric timing: SAC typically resolves first, while SPOC silencing requires additional time even after proper spindle alignment is achieved. Explicit delay terms are essential to recapitulate this experimentally observed ordering in unified SAC–SPOC models.

## 3. From ODEs to DDEs: Mathematical Background

Classical models of mitotic checkpoints are predominantly formulated as systems of ordinary differential equations (ODEs), in which the rate of change of each variable depends only on its current state. This framework has underpinned influential SAC and cell-cycle models, clarifying how nonlinear feedback, ultrasensitivity, and bistable switching shape checkpoint control [9,17,18,19,20,21,22]. However, ODEs assume effectively instantaneous biochemical responses and therefore cannot represent the experimentally observed molecular, spatial, and mechanochemical delays reviewed in the previous section.

**Figure 3:**
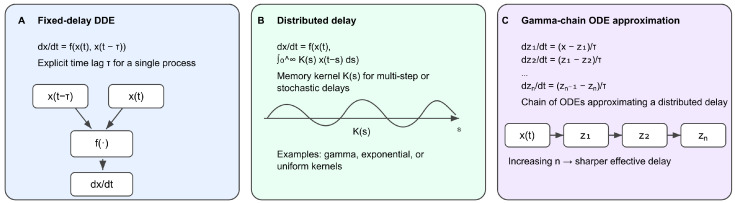
Mathematical representations of delays used in checkpoint modeling. (**A**) Discrete fixed-delay formulation in which the rate of change depends on both the current and delayed state X(t−τ). (**B**) Distributed-delay formulation using a memory kernel K(s) to weight a continuum of past states. (**C**) Gamma-chain ODE approximation, where sequential compartments $z_{1},\ldots ,z_{n}$ emulate a distributed delay with tunable variance. These formulations form the core mathematical tools used throughout SAC and SPOC delay modeling.

Delay differential equations (DDEs) generalize ODEs by allowing dependence on past states X(t−τ), providing a natural mathematical language for modeling multi-step assembly, spatial redistribution, transport processes, and delayed feedback [24,25,26,27,35,36]. DDEs therefore enable a more realistic treatment of checkpoint dynamics in the presence of finite processing times. Representations of delay-based formulations in checkpoint modeling are shown in Figure 3.

**Figure 4:**
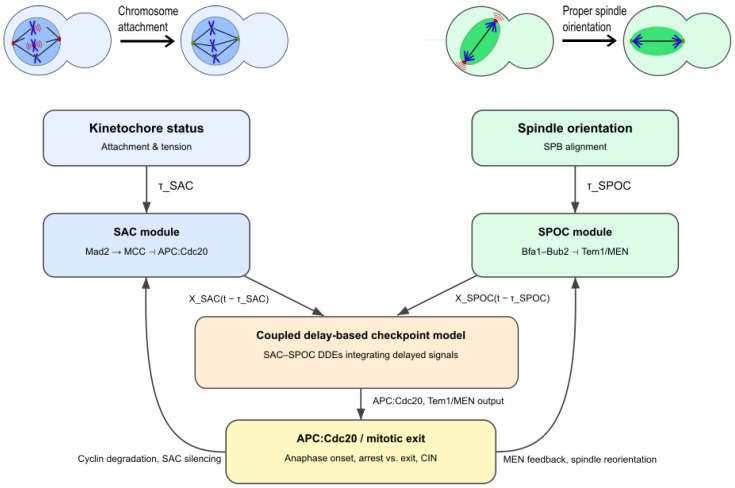
Integrated delay-based SAC–SPOC model architecture. Kinetochore attachment and tension feed the SAC module through the delay $\tau_{\mathrm{SAC}}$, whereas spindle orientation and SPB alignment feed the SPOC module through $\tau_{\mathrm{SPOC}}$. Both delayed signals converge in a coupled delay differential equation (DDE) framework regulating APC:Cdc20 activity and mitotic exit, with feedback from cyclin degradation and MEN reorientation closing the control loop.

Figure 4 provides a unified delay-based SAC–SPOC model architecture, linking attachment, tension, and spindle-orientation cues through delay terms $\tau_{\mathrm{SAC}}$ and $\tau_{\mathrm{SPOC}}$ within a coupled DDE framework.

### 3.1. Classical ODE Checkpoint Models

Early ODE models by Novak, Tyson, and co-workers demonstrated that feedback loops and covalent modification cycles can generate switch-like transitions in cell-cycle progression [17,18]. In the SAC, Doncic et al. showed that nonlinear interactions between MCC, APC/C, and regulatory feedback can produce ultrasensitive, bistable responses [19]. Subsequent ODE formulations incorporated Aurora B regulation, explicit kinetochore attachment states, and multi-step MCC assembly [6,9,20,21]. Minimal ODE SAC models, such as Henze et al. [22,37], reduce checkpoint regulation to a small set of effective species while retaining its bistable structure. SPOC modules based on Bfa1–Bub2–Tem1 have been treated similarly [3]. Although successful, these models represent delays implicitly via chains of intermediates or effective rates rather than explicitly.

### 3.2. Fixed-Delay DDE Formulations

A DDE extends an ODE by incorporating a discrete delay:

$$\frac{dX(t)}{dt}=F\left(X(t),X(t-\tau )\right),\;\tau >0.$$

Here, X(t) denotes the system state at time *t*, *F* is a nonlinear function of the current and delayed states, and τ>0 is the fixed delay parameter.

For example, coarse-grained MCC dynamics can be modeled as:

$$\frac{d\;\mathrm{MCC}(t)}{dt}=k_{f}\;u\left(t-\tau \right)-k_{d}\;\mathrm{MCC}\left(t\right),$$

where u(t) encodes kinetochore attachment or tension. Here, τ represents recruitment, conformational conversion, assembly, and transport delays.

Even small delay-driven systems can exhibit qualitatively new behaviors. Classical work by Mackey and Glass showed that increasing τ can induce oscillations and even chaos in nonlinear feedback systems [24]. Modern DDE theory provides precise characterizations of Hopf bifurcations, periodic orbits, and complex attractors in delay-driven systems [25,26,27]. Applied to SAC and SPOC, these analyses clarify how increasing effective delays in MCC production, APC/C inhibition, or Tem1 activation can generate oscillatory or irregular checkpoint responses.

### 3.3. Distributed Delays and Memory Kernels

Biological processes such as MCC assembly, spatial transport, and phosphorylation cascades occur with variable durations and are not well captured by a single discrete delay. They can instead be represented using *distributed* delays:

$$\frac{dX(t)}{dt}=f\left(X(t)\right)+\int_{0}^{\infty }K\left(s\right)\;g\left(X(t-s)\right)\;ds,$$

where K(s) is a delay kernel.

Gamma and Erlang kernels arise naturally from cascades of first-order processes and are widely used in physiological modeling [24,25]. Distributed delays encode a weighted dependence on past history rather than a single past time point, aligning closely with how SAC and SPOC integrate attachment, tension, and orientation signals over extended timescales [1,3,10].

### 3.4. Chain-of-ODE Approximations

Distributed delays can be approximated by a chain of ODE compartments. For a gamma-distributed delay with shape parameter *n* and mean τ,

$$\frac{dY_{1}}{dt}=\alpha \left(X\left(t\right)-Y_{1}\right),\;\frac{dY_{i}}{dt}=\alpha \left(Y_{i-1}-Y_{i}\right),\;i=2,\ldots ,n,$$

where α=n/τ. The final compartment $Y_{n}\left(t\right)$ behaves as a smoothed, delayed version of X(t).

This approach preserves Markovian structure, allows use of standard ODE solvers and continuation tools, and provides a direct biological interpretation representing sequential assembly or transport steps. Recent reaction network theory offers tools for analyzing such augmented systems, including persistence and invariant subspaces [30,31]. Hybrid models combining explicit discrete delays with chain approximations provide a flexible compromise between mechanistic realism and mathematical tractability.

In summary, DDEs extend ODE-based checkpoint formulations by explicitly representing the finite time lags inherent to SAC and SPOC biology. They reveal dynamical regimes—such as delay-induced oscillations, memory effects, and irregular or complex attractors [38]—that are inaccessible or obscured in purely instantaneous frameworks. The next sections review DDE-based checkpoint models and illustrate how explicit delays reshape mitotic control. Key analytical and numerical methods for studying delay-driven SAC and SPOC dynamics are summarized in Box 2.

Box 2 **Mathematical Toolbox for Delay-Based Checkpoint Models.**

Delay differential equations (DDEs) require specialized analytical and numerical tools:

- **Characteristic Equation:** Equilibrium stability follows from solving
  $$\lambda =f_{x}+f_{y}e^{-\lambda \tau },$$
  whose roots determine decay, oscillations, or growth of perturbations.
- **Hopf Bifurcations:** Increasing a delay τ can destabilize a steady state and give rise to periodic orbits.
- **DDE Continuation (e.g.,****DDE-Biftool****):** Enables tracking equilibria, periodic solutions, turning points, and codimension-2 bifurcations [39]. Essential for systematic exploration of SAC and SPOC delay dynamics.
- **Distributed Delays:** Gamma/Erlang kernels capture variability in MCC formation, APC/C inhibition, or Tem1 regulation.
- **Gamma-Chain Approximation:** Converts distributed delays into ODE chains with mean τ and variance $\tau^{2}/n$.
- **History Functions:** Initial conditions must specify the past trajectory over the interval $\left[t-\tau_{\max},0\right]$.
- **Noise–Delay Interactions:** Delays can filter or amplify stochastic fluctuations, contributing to observed variability in mitotic timing.

These tools form the foundation for analyzing delay-driven SAC and SPOC dynamics.

### 3.5. Numerical Solution Strategies for ODE and DDE Checkpoint Models

Numerical solution of checkpoint models depends on the underlying mathematical formulation. Classical ODE-based models can be integrated using standard explicit or implicit solvers (e.g., Runge–Kutta or BDF methods), with numerical stiffness often necessitating adaptive step-size control.

Fixed-delay DDE models additionally require specification of a history function over a finite interval preceding the start of integration. Dedicated solvers, such as dde23 in MATLAB R2024b or analogous tools in Python, explicitly account for this history dependence while enforcing continuity at delay boundaries.

Distributed-delay models may be treated directly using quadrature-based approaches or approximated by gamma-chain ODE systems, enabling the use of standard ODE solvers at the cost of increased dimensionality.

Across all formulations, numerical stability and accuracy depend on time-scale separation, delay magnitude, and nonlinearity strength. Practical implementations therefore require careful solver selection, appropriate tolerances, and validation against reduced or limiting cases.

## 4. DDE-Based Models of the Spindle Assembly Checkpoint

Mathematical models of the spindle assembly checkpoint (SAC) span detailed biochemical ODE systems to highly reduced bistable modules [6,9,17,19,20,21,22]. These ODE formulations encode delays only implicitly through cascades of intermediates or effective rates. Delay differential equation (DDE) extensions make such time lags explicit, providing a natural setting to examine how delayed MCC production, APC/C inhibition, and tension sensing reshape SAC dynamics. Below, key SAC modeling frameworks are reviewed, and it is shown how each can be interpreted or extended using DDE concepts.

### 4.1. Minimal Bistable SAC Modules with Explicit Delays

A recurring insight from SAC modeling is that checkpoint activation and silencing operate as a switch. ODE work by Doncic et al. demonstrated how nonlinear interactions between MCC, APC/C, and feedback loops generate ultrasensitive, bistable behavior [19]. Henze et al. distilled this structure into a minimal module involving effective MCC, active APC:Cdc20, and an upstream signal [22].

Delays can be introduced naturally into such reduced systems. For instance, MCC production can depend on past kinetochore signals, while APC/C inhibition can depend on earlier MCC levels:

$$\frac{d\;\mathrm{MCC}(t)}{dt}=f\left(u\left(t-\tau_{1}\right),\mathrm{MCC}\left(t\right)\right),\;\frac{d\;\mathrm{APC}:\mathrm{Cdc}20(t)}{dt}=g\left(\mathrm{APC}:\mathrm{Cdc}20\left(t\right),\mathrm{MCC}\left(t-\tau_{2}\right)\right).$$

Here u(t) encodes attachment or tension, and $\tau_{1},\tau_{2}$ represent effective recruitment, assembly, or transport times. DDE theory predicts that increasing delays in such feedback loops can induce Hopf bifurcations, oscillations, and even complex dynamics in strongly nonlinear regimes [24,25,26,27], offering mechanistic interpretations of irregular MCC or APC:Cdc20 fluctuations that are difficult to capture with instantaneous models.

### 4.2. Template-Based Mad2 Activation and Catalytic MCC Formation

Another class of SAC models centers on the molecular details of template-mediated Mad2 activation. Experiments show that Mad1–Mad2 complexes convert O-Mad2 to C-Mad2, which then participates in MCC assembly [4,5,6,10,14]. ODE models encode these steps as networks of binding and conformational reactions [6,9,20,21].

Because template-based activation involves multiple steps with variable durations, DDE formulations treat MCC production as a delayed or distributed-delay process:

$$\frac{d\;\mathrm{MCC}(t)}{dt}=k_{f}\;\int_{0}^{\infty }\;K_{Mad2}\left(s\right)\;u\left(t-s\right)\;ds-k_{d}\;\mathrm{MCC}\left(t\right),$$

where $K_{Mad2}\left(s\right)$ captures variability in activation and assembly times. This reflects the biological observation that MCC production integrates kinetochore states over time rather than responding to a single instant [6,10]. DDE variants of Mad2-centric models therefore illuminate how the timescale and variability of catalytic activation influence the robustness and responsiveness of SAC signaling.

### 4.3. Attachment- and Tension-Controlled SAC Models with Effective Delays

Several SAC models explicitly represent kinetochore attachment and tension regulation. Mistry et al. incorporated Aurora B-dependent phosphorylation to link tension to SAC protein recruitment and silencing [8,9]. Other frameworks emphasize error correction–attachment kinetics [28] or spatial aspects such as transport of checkpoint regulators [1,7,10,11].

In these models, delays arise from tension buildup, propagation of phosphorylation states, and transport from kinetochore to cytoplasm. For example, the time required for mechanical stabilization and phosphatase action can be modeled as a delayed silencing input $\tau_{\mathrm{tension}}$. Similarly, the influence of local kinetochore status on global APC/C activity can be delayed to represent microtubule-based transport. Varying such delays within DDE frameworks enables a systematic exploration of the trade-off between rapid SAC silencing and robustness to transient tension fluctuations.

### 4.4. Towards Unified Bistable Template SAC Models with Delays

Minimal feedback modules, Mad2-based catalytic activation, and attachment/tension models emphasize different mechanistic layers of the SAC [6,7,9,10,19,22]. A natural direction is to unify these perspectives in *bistable template* SAC models with explicit delays assigned to key processes such as Mad2 activation, MCC production, APC:MCC turnover, and tension-dependent silencing.

In such unified models, delays correspond directly to measurable biochemical and mechanochemical timescales. By varying these delays and analyzing the resulting bifurcation structure with DDE tools [24,25,26,27], one can map how subprocess timing shapes global SAC behavior—including bistability, oscillations, long transients, and more complex temporal patterns in MCC and APC:Cdc20 dynamics. Thus, DDE-based SAC formulations extend classical ODE models by explicitly capturing the intrinsic temporal structure of checkpoint control.

## 5. DDE-Based Models of the Spindle Position Checkpoint

While the SAC monitors kinetochore attachment and tension, the spindle position checkpoint (SPOC) ensures that chromosome segregation and cytokinesis occur along the correct division axis. SPOC has been studied most extensively in asymmetrically dividing cells such as budding yeast, where misaligned spindles can produce lethal missegregation if mitotic exit occurs prematurely. Functionally, SPOC acts as a late mitotic safeguard by coupling spindle position to the mitotic exit network (MEN) through the Bfa1–Bub2 complex and the small GTPase Tem1 [3].

Although fewer mathematical models exist for SPOC than for the SAC, the same delay-based reasoning applies. Spatial sensing of spindle position, relocalization of regulatory complexes, and multi-step control of Tem1 and MEN all introduce intrinsic time lags that can be represented naturally using delay differential equations [3,17].

### 5.1. Minimal SPOC Modules and Effective Delays

At a coarse level, SPOC can be represented as a minimal inhibitory module in which Bfa1–Bub2 suppresses Tem1 activity until the spindle becomes properly aligned. ODE-based formulations capture the logical behavior: misalignment maintains high Bfa1–Bub2 activity and low Tem1, while correct alignment eventually permits Tem1 activation and mitotic exit [3].

Introducing explicit delays highlights the finite times needed for positional sensing and relocalization. A simple DDE representation is

$$\frac{d\;\mathrm{Tem}1(t)}{dt}=F_{\mathrm{SPOC}}\left(a\left(t-\tau_{\mathrm{pos}}\right),Bfa1Bub2\left(t\right),\mathrm{Tem}1\left(t\right)\right),$$

where a(t) represents spindle alignment, Bfa1Bub2(t) encodes inhibition, and $\tau_{\mathrm{pos}}$ aggregates the time for alignment cues to translate into effective Tem1 activation. Increasing $\tau_{\mathrm{pos}}$ prolongs mitotic arrest even after correct spindle positioning, introducing a SPOC “memory’’ analogous to SAC memory from MCC/APC:Cdc20 turnover delays.

### 5.2. Bfa1–Bub2 Regulation, Cdc5 Feedback, and Multi-Step SPOC Delays

SPOC regulation involves phosphorylation and relocalization events controlled by the Polo-like kinase Cdc5. Cdc5-mediated phosphorylation inactivates or relocalizes Bfa1–Bub2, thereby permitting Tem1 activation once alignment is correct [3]. These steps unfold over finite timescales: Cdc5 must accumulate, substrates must be phosphorylated, and downstream MEN components must respond.

DDE formulations introduce explicit delays for these processes:

$$\frac{d\;Bfa1Bub2(t)}{dt}=H\left(Bfa1Bub2\left(t\right),\mathrm{Cdc}5\left(t-\tau_{\mathrm{Cdc}5}\right)\right),$$

where $\tau_{\mathrm{Cdc}5}$ captures the lag between upstream cues (or SAC satisfaction) and the effective action of Cdc5 on Bfa1–Bub2. Together, $\tau_{\mathrm{pos}}$ and $\tau_{\mathrm{Cdc}5}$ determine how quickly SPOC is silenced after correct alignment and how robustly the system resists transient misalignment.

### 5.3. SAC–SPOC Ordering and Asymmetric Delays

A consistent experimental observation is that SAC resolves before SPOC: cells first achieve proper kinetochore attachment and tension, then finalize spindle alignment and exit mitosis [3,10,40,41]. Unified models interpret this ordering as a consequence of distinct delay scales. SAC processes—Mad2 activation, MCC assembly, APC/C inhibition—typically operate on shorter effective delays, whereas SPOC processes—Bfa1–Bub2 relocalization, Cdc5-mediated phosphorylation, and MEN activation—introduce additional multi-step delays.

DDE frameworks make this explicit by assigning separate delay parameters, such as $\tau_{\mathrm{SAC}}$ for MCC/APC dynamics and $\tau_{\mathrm{SPOC}}$ for positional sensing and Tem1/MEN regulation. Regimes with $\tau_{\mathrm{SPOC}}>\tau_{\mathrm{SAC}}$ reproduce the experimentally observed ordering and allow exploration of perturbations that disrupt it, such as mutations affecting transport or kinase activity.

### 5.4. Towards Integrated SAC–SPOC DDE Frameworks

SPOC should be understood in the context of the broader mitotic control network. Integrated SAC–SPOC models describe how attachment, tension, spindle position, and CDK activity collectively govern the decision to remain arrested in mitosis or proceed into anaphase and cytokinesis [3,17]. DDEs naturally encode the multiple overlapping delays within each checkpoint and their crosstalk.

Hybrid models incorporating explicit delays for key processes and chain-of-ODE approximations for distributed delays provide a practical balance between realism and tractability. Recent advances in reaction-network theory and delay systems [26,27,30,31] support the analysis of such frameworks, including persistence properties, invariant sets, and delay-induced bifurcations.

In summary, although explicit SPOC DDE models are less developed than SAC models, the same principles apply: positional sensing, relocalization, and kinase-mediated feedback introduce intrinsic delays that strongly shape mitotic timing and robustness. Making these delays explicit is essential for an integrated, mechanistic understanding of how SAC and SPOC jointly maintain fidelity during mitosis.

## 6. How Delays Shape Mitotic Dynamics

Explicit time delays fundamentally reshape the dynamics of mitotic checkpoints. Even simple delayed feedback systems can exhibit oscillations, bistability, long transients, and chaotic behavior [24,25,26,27]. In SAC and SPOC, such delay-driven phenomena translate into differences in how cells arrest, maintain, and terminate checkpoint signaling. DDE-based models therefore clarify not only whether a checkpoint is active, but how it fluctuates, how long memory persists, and how sensitive the system is to intrinsic and extrinsic noise.

### 6.1. Delay-Induced Oscillations and Transient Cycling

A hallmark of delayed negative feedback is the emergence of oscillations via Hopf bifurcations. When a delay exceeds a critical threshold, a stable state can lose stability and give rise to sustained periodic behavior [24,25]. In SAC-related DDE models, such oscillations may appear as cyclic variations in MCC concentration, APC:Cdc20 activity, or tension-dependent readouts. These represent repeated strengthening and weakening of checkpoint signaling even without new attachment errors, driven purely by delayed biochemical or mechanical feedback.

In models coupling SAC with attachment and tension dynamics, delay-induced oscillations may correspond to repeated attempts at error correction: delayed feedback produces overshoot–undershoot cycles before the system settles into arrest or satisfaction [3,9,22,28]. Such transient oscillations are difficult to obtain with purely instantaneous ODEs unless many intermediates are added. DDEs instead provide a parsimonious explanation for experimentally observed fluctuations in checkpoint markers and the variability of mitotic duration [1,12,13,42,43].

### 6.2. Delays, Bistability, and Checkpoint Memory

Bistability underlies robust checkpoint control: cells either remain arrested or commit to anaphase with limited intermediate behavior [17,19,22]. Classical ODE models capture this through nonlinear feedback between MCC, APC/C, and cell-cycle regulators. Adding delays alters the structure and robustness of these bistable regimes.

Delays effectively add *memory*, because the dynamics depend on a segment of past states rather than on a single point. This can sharpen or weaken basin boundaries, generate long plateaus, or prolong near-arrested dynamics even after upstream conditions change [24,25]. In SAC models, delays in MCC production or APC:MCC disassembly can prolong the time required to silence the checkpoint, producing a mechanistic form of checkpoint memory that safeguards against premature anaphase onset [3,22].

SPOC exhibits analogous delay-induced memory: Tem1/MEN activation often lags behind final spindle alignment due to positional sensing and Cdc5-dependent relocalization [3]. In integrated SAC–SPOC DDE models, differences in delay scales naturally explain the experimentally observed ordering in which SAC resolves before SPOC [1,3,10].

### 6.3. Delay-Induced Complexity and Chaotic Regimes

For sufficiently large delays or strong nonlinearities, DDEs can exhibit period-doubling cascades and chaos [24,25,26,27]. Although chaos has not been demonstrated in fully realistic SAC or SPOC models, theoretical considerations suggest that the combination of delayed feedback, sharp nonlinear activation (e.g., APC/C switching), and coupled checkpoints could support irregular, aperiodic behavior in certain parameter regimes.

Biologically, such complex dynamics might correspond to pathological conditions in which checkpoint signaling becomes unstable, producing irregular fluctuations in cyclin levels, APC/C activity, or checkpoint markers. These features resemble aspects of chromosomal instability in cancer cells [1,44,45,46,47]. Even if full chaos is rare physiologically, the sensitivity of delay-driven systems emphasizes the need for finely tuned timing within checkpoint networks.

### 6.4. Delays, Noise, and Variability in Mitotic Timing

Mitotic checkpoints operate under substantial intrinsic noise due to low copy numbers, stochastic binding events, and fluctuating forces [9,20,21,28,48]. Delays interact with this noise in complex ways. In some regimes, delayed feedback filters high-frequency fluctuations; in others, it amplifies noise, increasing variability in checkpoint outputs and mitotic duration—effects related to stochastic or coherence resonance [24,25].

DDE-based stochastic models allow systematic exploration of these effects. For the SAC, such analyses help explain why genetically identical cells show broad distributions of mitotic timing and diverse responses to spindle poisons [1,12,13,49]. In SPOC, delays and noise jointly determine the reliability with which misaligned spindles are detected before mitotic exit [3].

### 6.5. Bifurcation-Based Classification of Delay Effects

**Figure 5:**
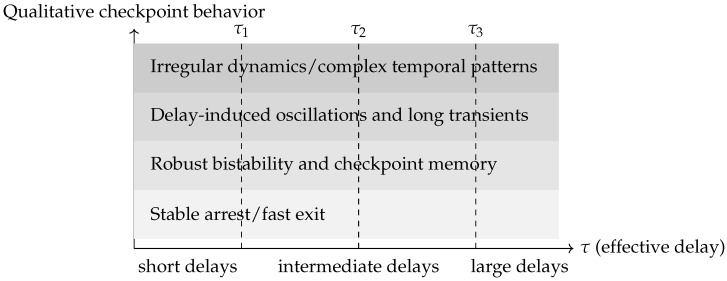
Conceptual delay–regime map for mitotic checkpoint dynamics. The horizontal axis represents an effective delay parameter τ (e.g., combining Mad2 activation, MCC assembly, APC/C inhibition, transport, or Tem1/MEN regulation), while the shaded bands summarize qualitatively distinct dynamical regimes. For small delays, SAC and SPOC settle into stable arrest or timely exit. Increasing τ supports robust bistability and checkpoint memory, followed by delay-induced oscillations and prolonged transients via Hopf bifurcations. For sufficiently large delays and strong nonlinear feedback, the system may enter regimes with irregular or complex temporal patterns. The diagram is schematic and does not correspond to a specific parameter set.

Bifurcation analysis provides a unifying framework for organizing delay-induced phenomena. Treating delays as bifurcation parameters and applying continuation tools for DDEs [26,27] allows one to track equilibria, periodic orbits, and stability boundaries as time lags vary. This reveals critical thresholds at which arrested states lose stability, oscillations emerge, or long transients appear. A conceptual overview of these delay-controlled regimes is shown in Figure 5.

For minimal SAC and SPOC modules, such diagrams map the regions of robust arrest, timely exit, oscillatory dynamics, and more complex regimes. For larger models, bifurcation-based screening identifies key delay combinations that govern qualitative behavior, guiding both experiments and systematic model reduction [17,22,30,31]. Delays thus serve as central organizing parameters that structure the space of possible checkpoint behaviors.

Taken together, explicit delays are a primary source of dynamical richness in mitotic checkpoint networks. They underlie oscillations, memory, variability, and potential complex dynamics in SAC and SPOC, and must be represented explicitly to achieve a mechanistically faithful picture of mitotic control. The next section examines how delays interact with spatial and mechanical aspects of checkpoint regulation and how mechanochemical DDE models can connect signaling to forces and geometry.

### 6.6. Illustrative Example: Delay-Induced Hopf Bifurcation in a Minimal Model

**Figure 6:**
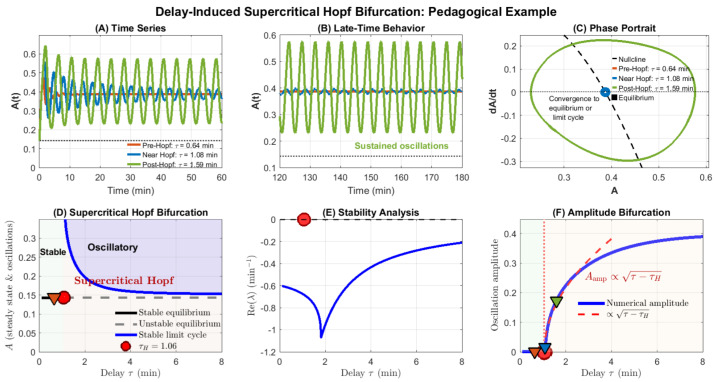
**Delay-induced supercritical Hopf bifurcation in a minimal delayed negative feedback model.** (**A**) Time series for three representative delays: pre-Hopf (τ=0.64 min, orange), near-Hopf (τ=1.08 min, blue), and post-Hopf (τ=1.59 min, green), shown over a restricted early-time window to improve visual clarity. For $\tau <\tau_{H}$ the trajectory relaxes monotonically to the equilibrium $A^{*}$, whereas for $\tau >\tau_{H}$ sustained oscillations emerge. (**B**) Late-time zoom (120–180 min) showing convergence to the fixed point (orange, blue) versus a stable limit cycle (green). (**C**) Phase portrait (*A* vs. dA/dt) illustrating the stable fixed point (black square), the nullcline (black dashed line), and the emergence of closed orbits for $\tau >\tau_{H}$ (green), highlighting convergence to the equilibrium or a stable limit cycle. Pre-Hopf trajectories spiral inward, while post-Hopf trajectories evolve onto a stable limit cycle. (**D**) **Hopf bifurcation diagram**: The equilibrium $A^{*}$ (black line) is stable for $\tau <\tau_{H}\approx 1.06$ min and unstable for $\tau >\tau_{H}$ (gray dashed). Beyond the critical delay, a branch of stable limit cycles (blue envelope) appears, marking a *supercritical* Hopf bifurcation. Colored triangles indicate the three simulated delay values used in Panels (**A**)–(**C**). (**E**) Stability analysis of the linearized DDE: the real part of the dominant eigenvalue Re(λ) crosses zero at the theoretical Hopf point (red marker), separating the stable regime (Re(λ)<0) from the oscillatory regime. (**F**) **Amplitude bifurcation diagram**: Numerical oscillation amplitude (blue) grows continuously from zero for $\tau >\tau_{H}$, consistent with a supercritical Hopf bifurcation. A square-root scaling curve ($\propto \sqrt{\tau -\tau_{H}}$, red dashed) fits the initial growth regime. Triangles mark amplitudes corresponding to the simulated delays. Model parameters: $k_{\mathrm{act}}=0.5$ $\min^{-1}$, $k_{\mathrm{inh}}=3.0$ $\min^{-1}$, Hill coefficient n=4, half-saturation constant K=0.5.

To concretely demonstrate how time delays induce Hopf bifurcations and generate oscillatory checkpoint dynamics, a minimal delayed negative feedback model is analyzed. This pedagogical example distills the essential mechanism by which delays in SAC and SPOC signaling can destabilize checkpoint silencing. A complete bifurcation and stability analysis of this model is shown in Figure 6, which illustrates the emergence of oscillations through a supercritical Hopf bifurcation.

#### 6.6.1. Model Formulation

Consider a single-variable system representing a coarse-grained checkpoint regulator (e.g., active APC:Cdc20) with delayed nonlinear negative feedback:

(1) $$\frac{dA(t)}{dt}=k_{\mathrm{act}}\left(1-A\left(t\right)\right)-k_{\mathrm{inh}}\;A\left(t\right)\;f\left(A(t-\tau )\right),$$

where A(t) is the normalized regulator concentration, $k_{\mathrm{act}}$ represents spontaneous activation, $k_{\mathrm{inh}}$ quantifies inhibition strength, τ is the delay, and f(A) is a Hill-type nonlinear feedback function:

(2) $$f\left(A\right)=\frac{A^{n}}{K^{n}+A^{n}},$$

with Hill coefficient n=4 and half-saturation constant K=0.5. This structure captures delayed auto-inhibition analogous to MCC-mediated APC:Cdc20 suppression or Bfa1–Bub2 regulation of Tem1.

The system possesses a delay-independent equilibrium $A^{*}=k_{\mathrm{act}}/\left(k_{\mathrm{act}}+k_{\mathrm{inh}}\right)$. Linear stability analysis around $A^{*}$ yields a characteristic equation of the form λ+a+bexp(−λτ)=0, where *a* and *b* depend on the linearized feedback strength. For small τ, $A^{*}$ is stable; as τ increases past a critical value $\tau_{\mathrm{Hopf}}$, the leading eigenvalue crosses the imaginary axis, giving rise to a Hopf bifurcation.

#### 6.6.2. Bifurcation Analysis and Dynamical Regimes

Figure 6 presents a comprehensive bifurcation analysis. For the parameter set $k_{\mathrm{act}}=0.5$, $k_{\mathrm{inh}}=3.0$ $\min^{-1}$, the critical delay is identified as $\tau_{\mathrm{Hopf}}\approx 1.17$ min.

This numerical example is intended as a representative and illustrative demonstration of delay-induced bifurcation behavior, rather than an exhaustive simulation of all checkpoint models discussed in this review.

For $\tau <\tau_{\mathrm{Hopf}}$ (pre-Hopf regime), the system exhibits stable equilibrium behavior: small perturbations decay monotonically or via damped oscillations (Figure 6A, orange trace). The phase portrait shows trajectories spiraling inward to the fixed point (Figure 6C, orange).

At $\tau =\tau_{\mathrm{Hopf}}$, a supercritical Hopf bifurcation occurs. The equilibrium loses stability, and a family of stable limit cycles emerges. For $\tau >\tau_{\mathrm{Hopf}}$ (post-Hopf regime), the system exhibits sustained periodic oscillations whose amplitude grows continuously with increasing delay (Figure 6A,B, green trace). The phase portrait reveals closed orbits (limit cycles) of increasing radius (Figure 6C, green).

The Hopf bifurcation diagram (Figure 6D) captures this transition: the black line represents the equilibrium $A^{*}$, which is stable (solid) for $\tau <\tau_{\mathrm{Hopf}}$ and unstable (dashed) for $\tau >\tau_{\mathrm{Hopf}}$. The blue dashed curves show the maximum and minimum values of the emerging limit cycle, forming an envelope that grows smoothly from the bifurcation point. This characteristic “pitchfork” structure is the hallmark of supercritical Hopf bifurcation.

Stability analysis (Figure 6E) confirms that Re(λ) crosses zero at $\tau_{\mathrm{Hopf}}$, separating the stable region (green shading, Re(λ)<0) from the oscillatory region (Re(λ)>0). The amplitude bifurcation diagram (Figure 6F) provides the clearest visualization: oscillation amplitude is zero for $\tau <\tau_{\mathrm{Hopf}}$ and grows monotonically for $\tau >\tau_{\mathrm{Hopf}}$, demonstrating the smooth birth of oscillations characteristic of supercritical bifurcations. Although derived from a minimal delayed negative feedback model, the bifurcation structure, oscillation onset, and delay-induced memory effects illustrated here extend qualitatively to higher-dimensional SAC and SPOC models incorporating additional biochemical or mechanochemical detail.

#### 6.6.3. Implications for Checkpoint Dynamics

This minimal model illustrates several key principles relevant to SAC and SPOC:

1. **Delay-induced instability:** Even simple delayed negative feedback can destabilize steady states that are stable in the absence of delays, generating sustained oscillations.
2. **Threshold behavior:** The Hopf bifurcation defines a critical delay threshold $\tau_{\mathrm{Hopf}}$. Biological delays below this threshold yield robust, monotonic checkpoint silencing, whereas delays exceeding $\tau_{\mathrm{Hopf}}$ produce oscillatory or irregular dynamics.
3. **Amplitude scaling:** Post-Hopf oscillation amplitude increases with delay, suggesting that cells with longer transport times, slower MCC turnover, or more distributed assembly processes may exhibit greater temporal variability in checkpoint outputs.
4. **Memory and history dependence:** Delay-driven oscillations reflect the system’s dependence on its past trajectory over an interval [t−τ,t], providing a mechanistic basis for checkpoint memory effects observed experimentally [1,28].

In the context of mitotic checkpoints, such oscillations may underlie observed cell-to-cell variability in mitotic timing [12], prolonged mitotic arrest in response to spindle poisons [50], or irregular MCC/APC:Cdc20 dynamics during error correction [15]. While full SAC and SPOC models incorporate additional regulatory layers—multi-step MCC assembly, spatial compartmentalization, mechanochemical coupling—the core delay-induced bifurcation mechanism demonstrated here remains central to their dynamical behavior.

Importantly, this example highlights the value of DDE-based modeling: capturing the Hopf bifurcation and resulting limit cycles within a purely ODE framework would require either ad hoc chains of many intermediate variables or phenomenological oscillator terms, obscuring the mechanistic role of time delays. DDEs, in contrast, make the delay explicit and connect it directly to measurable biological timescales, enabling principled exploration of how subprocess timing shapes checkpoint control.

## 7. Integrating Mechanics, Spatial Dynamics, and Delays

Mitotic checkpoints are inherently spatial and mechanochemical. Kinetochores, microtubules, spindle poles, and the cortex form a three-dimensional, force-bearing architecture in which signaling molecules are transported, recruited, and mechanically regulated [1,7,10]. Forces acting through complexes such as Ndc80 and KNL1 modulate phosphorylation states that determine checkpoint activity [8,10]. At the same time, key regulators must move between compartments with distinct biophysical constraints [11].

Delay differential equations (DDEs) provide a natural way to incorporate these finite mechanochemical and spatial timescales without resolving full spatial geometry. Mechanochemical–DDE frameworks bridge detailed spatial models and coarse-grained biochemical ones, capturing how delays in force buildup, transport, and relocalization shape the temporal behavior of the SAC and SPOC.

### 7.1. Force-Dependent Kinetics and Tension Buildup Delays

SAC signaling is tightly linked to the mechanical state of kinetochore–microtubule attachments. Force-dependent conformational changes in outer-kinetochore complexes, coupled with Aurora B kinase and phosphatase activity, determine whether phosphorylation promotes checkpoint activation or silencing [7,8,10]. Proper tension stabilizes attachments and biases the system toward SAC satisfaction; low tension maintains signaling [1,10].

Force does not equilibrate instantaneously. Microtubule dynamics, motor activity, and viscoelastic properties of chromatin introduce characteristic timescales for tension buildup and relaxation. Mechanochemical models therefore incorporate delays in tension-dependent reactions. In DDE form, a representative delayed tension effect is:

$$k_{\mathrm{off}}\left(t\right)=k_{\mathrm{off}}^{0}\;\Phi \left(F(t-\tau_{\mathrm{mech}})\right),$$

where F(t) is a coarse-grained tension variable, Φ a force-response function, and $\tau_{\mathrm{mech}}$ an effective mechanotransduction delay. Varying $\tau_{\mathrm{mech}}$ reveals how slowly or rapidly mechanical information is integrated and how this shapes the likelihood of premature SAC silencing.

### 7.2. Spatial Organization, Transport, and Compartmental Delays

Checkpoint regulators are spatially segregated. Kinetochores act as signal-generating hubs, while APC/C and many downstream components reside in the nucleoplasm or at distinct subcellular structures [1,7,10]. Proteins such as Mad2, BubR1, Bub3, and MCC must be recruited, activated, and transported over micrometer scales to influence APC/C. Dynein-mediated motion, diffusion, and active trafficking all contribute to spatial reorganization [11].

Resolving these processes requires spatially extended models (reaction–diffusion or particle-based). However, many of their dynamical consequences can be captured using *compartmental delays* that summarize transport and maturation times. For example:

$$\frac{d\;\mathrm{APC}:\mathrm{Cdc}20(t)}{dt}=G\left(\mathrm{APC}:\mathrm{Cdc}20\left(t\right),\mathrm{MCC}\left(t-\tau_{\mathrm{trans}}\right)\right),$$

where $\tau_{\mathrm{trans}}$ represents the delay between kinetochore signaling and cytosolic APC/C inhibition. Multi-compartment ODE models augmented with distributed or gamma-chain delays provide a natural intermediate between fully spatial and purely temporal descriptions [11,25,30,31].

### 7.3. Mechanochemical SPOC Control and Orientation Delays

The SPOC monitors spindle orientation through mechanical interactions between astral microtubules, spindle pole bodies, and cortical cues [3]. Bfa1–Bub2 and Tem1 localize to spindle poles, and their activities depend on mechanical alignment and spatial relocalization. These processes occur on finite timescales driven by microtubule forces, motor activity, and MEN signaling.

Explicit delays capture these mechanochemical features: delays in Bfa1–Bub2 phosphorylation, Tem1 activation, relocalization between poles, and MEN response. Such delayed interactions determine how quickly SPOC can be silenced after the spindle aligns and how robust the system is to transient misalignment.

### 7.4. Hybrid Mechanochemical DDE Frameworks

Purely DDE-based models and fully spatial mechanochemical models represent two ends of a modeling spectrum. Hybrid formulations combine explicit mechanical or spatial degrees of freedom (e.g., tension variables, spindle orientation angles) with delayed biochemical terms describing MCC, APC/C, Bfa1–Bub2, and Tem1 dynamics [3,9,17].

Recent advances in reaction-network theory identify persistent subspaces and invariant structures in such hybrid models [30,31], while DDE bifurcation tools enable joint exploration of mechanical and delay parameters [26,27]. This establishes a framework in which mechanical forces, spatial transport, and delayed biochemical signaling jointly determine checkpoint timing, robustness, and error-correction efficiency.

Taken together, mechanochemical and spatial delays are essential determinants of SAC and SPOC behavior. Integrating these features into DDE-based frameworks moves checkpoint modeling toward a unified, holistic description of mitosis that connects molecular interactions with emergent cellular dynamics. Key experimental methods for quantifying SAC and SPOC delays are summarized in Box 3.

Box 3 **Experimental Techniques for Quantifying Delays in Mitotic Checkpoints.**

Accurately parameterizing delays in SAC and SPOC requires experimental methods capable of resolving mechanical responses, transport times, and biochemical turnover:

- **Live-cell fluorescence imaging:** Measures recruitment, activation, and disassembly timescales for Mad2, BubR1, MCC, and APC/C.
- **FRET tension sensors:** Quantify tension buildup and relaxation at kinetochore complexes such as Ndc80 and KNL1.

- **FRAP and photoactivation:** Reveal turnover delays of checkpoint proteins under distinct attachment or tension states.
- **Optogenetic perturbations:** Provide precise temporal control of Aurora B activity, kinetochore recruitment, or spindle forces to measure mechanochemical response times.
- **Single-cell mitotic timing assays:** Quantify population-level delay distributions for SAC satisfaction and SPOC-controlled exit.
- **Fluorescent cyclin/securin degradation reporters:** Monitor APC/C activation and associated delays in SAC and SPOC signaling.
- **Spindle orientation tracking (SPOC):** High-resolution imaging of spindle pole bodies and cortical markers measures delays in Bfa1–Bub2 relocalization and Tem1/MEN activation.

These techniques provide the empirical foundation for parameterizing and validating mechanochemical DDE models.

## 8. Hybrid AI–DDE Modeling for Mitotic Checkpoints

Quantitative live-cell imaging, single-cell tracking, and high-throughput perturbations have prompted growing use of machine learning and AI in cell-cycle research. In parallel, mechanistic ODE-, DDE-, and reaction-network models remain indispensable for capturing regulatory topology, feedback logic, and biochemical and mechanochemical structure [3,17,18,22,30,31]. Hybrid AI–mechanistic approaches seek to combine these complementary strengths: mechanistic models provide structure and interpretability, while AI methods learn unknown interactions, infer parameters, and interface with high-dimensional data.

DDEs are especially well suited for hybrid frameworks because they explicitly encode history dependence. This mirrors experimental evidence that SAC and SPOC responses depend on the past evolution of attachment, tension, and spindle position over tens of minutes, not just instantaneous states [1,3,9,10,22,28]. AI can then be deployed to learn effective delay distributions, memory kernels, and history-dependent response functions consistent with mechanistic structure.

### 8.1. Learning Effective Delays and Memory Kernels

A natural hybrid strategy uses mechanistic DDEs as structural scaffolds, while AI methods estimate delay parameters and kernel shapes. Consider a distributed-delay formulation,

$$\frac{dX(t)}{dt}=f\left(X(t)\right)+\int_{0}^{\infty }K_{\theta }\left(s\right)\;g\left(X(t-s)\right)\;ds,$$

where $K_{\theta }\left(s\right)$ parameterizes the delay distribution. Gradient-based optimization, variational inference, or Bayesian approaches can fit θ to time-series imaging data, yielding effective delay distributions for MCC production, APC/C inhibition, or Tem1 activation [17,22,25].

More flexible variants represent $K_{\theta }\left(s\right)$, *f*, or *g* with neural networks constrained by biochemical structure. This allows the data to refine memory functions while preserving checkpoint topology and interpretability.

### 8.2. Neural DDE Surrogates and Model Reduction

Mechanochemical checkpoint models combining spatial structure, forces, and delayed biochemistry can be computationally demanding. Neural DDE surrogates provide reduced models that approximate the input–output behavior of detailed systems while retaining an internal delayed structure. These surrogates accelerate simulation, parameter exploration, and bifurcation analysis while maintaining mechanistic interpretability in terms of delays, feedback strengths, and effective reaction rates [17,22,30,31].

### 8.3. Data Assimilation and Reconstruction of Hidden Histories

DDEs depend on system history over an interval $\left[t-\tau_{\max},t\right]$, yet experiments often provide only partial observables (e.g., APC/C activity, MCC levels, spindle-orientation trajectories). Hybrid AI–DDE approaches can reconstruct missing histories by combining a mechanistic model with data-driven inference. Adjoint-based optimization, recurrent neural networks, or differentiable DDE solvers can infer unobserved attachment or tension histories that make model outputs agree with experimental data [24,25]. Such techniques are especially relevant for SAC and SPOC, where many mechanochemical variables are experimentally inaccessible.

### 8.4. Structure Discovery and Model Selection with Delays

AI can also aid discovery of mechanistic structure, including which processes carry delays and how these delays are distributed. Because multiple reaction-network topologies can reproduce similar behaviors, structure selection benefits from combining data-driven scoring with mechanistic constraints [30,31]. Hybrid approaches evaluate families of DDE models—differing in delay placement, kernel type, or feedback topology—against experimental datasets. Reaction-network criteria then filter out structurally implausible or nonpersistent models [17]. The result is an ensemble of interpretable mechanistic candidates consistent with available data.

### 8.5. Towards AI-Assisted Mechanochemical DDE Models

In this section, the term *AI-assisted* refers to a family of data-driven and hybrid computational approaches that complement mechanistic delay differential equation (DDE) models, rather than replacing them. These approaches include classical machine learning methods for parameter inference and regime classification, neural-network-based models (including neural delay differential equations) for surrogate modeling and accelerated simulation, symbolic regression techniques for discovering parsimonious delay-dependent interactions, and Bayesian or physics-informed learning frameworks for uncertainty quantification and constrained inference.

Overall, AI and DDEs are synergistic tools for modeling mitotic checkpoints. DDEs preserve the biological principle that SAC and SPOC integrate history, while AI enables efficient parameterization, model reduction, and data assimilation. In the context of SAC and SPOC, hybrid AI–DDE frameworks can:

- infer effective delays, memory kernels, and kinetic parameters for Mad2 activation, MCC assembly, APC/C inhibition, and Tem1/MEN regulation using machine learning or Bayesian inference methods,
- learn reduced or surrogate models, for example via neural networks or neural DDEs, that retain mechanistic meaning while enabling fast parameter scans,
- reconstruct unobserved mechanochemical histories from limited or noisy data through data assimilation or physics-informed learning approaches,
- and support structural and functional discovery in the space of delay-extended checkpoint architectures using symbolic regression or sparse model discovery techniques.

These capabilities will be essential for building quantitatively accurate, mechanistically interpretable models of mitotic control and for linking delay-based checkpoint models to experimental and therapeutic interventions.

## 9. Open Challenges in Delay-Based Checkpoint Modeling

Although delay-based models have deepened our understanding of SAC and SPOC regulation, major conceptual and technical challenges remain. These involve experimental quantification of delays, multi-delay inference, stochasticity, numerical complexity, and multi-scale integration. Addressing them is essential for fully leveraging DDEs in mitotic checkpoint research.

### 9.1. Measuring Intracellular Delays and Linking Them to Mechanisms

A key challenge is the quantitative determination of delays themselves. Live-cell imaging provides timing of MCC accumulation, APC:Cdc20 activity, and mitotic arrest, but disentangling the contributions of Mad2 activation, MCC assembly, transport, kinesin- and dynein-driven movement, force-dependent conformational changes, and APC/MEN remodeling is still difficult [1,9,10,12,13,28]. Similarly, SPOC delays arise from spindle realignment, Bfa1–Bub2 regulation, Tem1 activation, and MEN responses [3].

Progress requires combined experimental and modeling pipelines: quantitative imaging, mechanical measurements, targeted perturbations (e.g., kinase inhibition, transport disruption), and DDE-based inference techniques that map composite delays to specific mechanisms [17,22,25]. For human cells and disease contexts, such mechanistic mapping remains largely unexplored.

In addition to SAC and SPOC, anaphase onset is also regulated by surveillance mechanisms that monitor the completion of DNA strand passage reactions mediated by Topoisomerase II. Recent studies indicate that SUMOylation-dependent control of Topoisomerase II, together with downstream signaling involving Haspin kinase, Aurora B, and Mad2, contributes to delaying anaphase until chromosome disentanglement is complete. Although explicit modeling of this pathway lies beyond the scope of the present review, its incorporation into future delay-based checkpoint frameworks would be essential for capturing how multiple surveillance modules jointly coordinate mitotic progression [51].

An additional and compounding challenge is that intracellular delays are not universal, but depend strongly on cellular context. Checkpoint architecture, timing, and robustness differ between normal cells and genetically unstable or transformed cells. More than half of human cancers exhibit chromosome instability, often accompanied by mutations or dysregulation in SAC and SPOC components, including Mad2, BubR1, Aurora B, APC/C regulators, and mitotic kinases. As a result, delays measured in cancer cell lines may reflect altered checkpoint wiring rather than baseline physiological timing. This raises an important interpretational issue: quantitative delay measurements obtained outside of normal cellular contexts must be treated with caution when used to parameterize mechanistic models of checkpoint control. Distinguishing conserved, core delay mechanisms from disease-specific alterations remains an open challenge for both experiments and delay-based modeling.

### 9.2. Multi-Delay Systems and Identifiability

Realistic checkpoint models involve several delays—Mad2 activation, MCC assembly, APC/C inhibition, APC:MCC disassembly, tension buildup, transport, Tem1/MEN regulation—yet many of these delays cannot be uniquely inferred from standard observables. This creates a *multi-delay identifiability* challenge: determining which delays are distinguishable, which are redundant, and which require external constraints or mechanistic priors [24,25,26,27]. Developing identifiability frameworks for DDEs, together with perturbation designs that maximize information content, is essential to avoid overfitting delay-rich models [17,22,30,31].

### 9.3. Stochasticity, Noise, and Hybrid Stochastic–DDE Models

Early SAC and SPOC processes operate under strong stochasticity due to low copy numbers and discrete binding events [9,19,20,21,28]. Existing DDE models are predominantly deterministic and treat delays as fixed or smoothly distributed. Extending them to stochastic settings raises difficult questions: how to model fluctuations in delayed reactions, how to define stochastic history dependence, and how to simulate such systems efficiently.

Hybrid stochastic–DDE frameworks could capture interactions between noise and delayed feedback, clarifying variability in mitotic timing, checkpoint escape, and chromosomal instability [1,10,44,45]. However, numerical methods for stochastic DDEs remain computationally expensive, and checkpoint-specific approximations are still lacking.

### 9.4. Numerical Stiffness, Scalability, and Bifurcation Analysis

Delay-based checkpoint models with mechanochemical coupling and multiple time scales are often stiff and high-dimensional [3,17,22]. In this context, *numerical stiffness* arises from the coexistence of fast biochemical reactions (e.g., phosphorylation, binding/unbinding) and slow delayed processes (e.g., MCC accumulation, transport, tension maturation), leading to widely separated time scales within a single model. Stiffness imposes severe constraints on time stepping, often requiring implicit solvers, adaptive step-size control, or specialized stiff DDE integrators to ensure numerical stability and accuracy.

Although numerical continuation and stability tools for DDEs are advancing [26,27], their application to detailed SAC–SPOC models remains challenging. Scalability is a major limitation: as the number of delayed processes, feedback loops, and mechanochemical variables increases, the effective dimensionality of the system grows substantially due to the infinite-dimensional history space associated with delays. This growth impacts both direct simulation and bifurcation analysis, making parameter sweeps, continuation, and sensitivity analysis computationally demanding even for moderately sized checkpoint models [52].

Future progress will require: (i) efficient stiff DDE solvers for biochemical networks, (ii) scalable bifurcation methods for systems with several delays, and (iii) principled model reduction that preserves delay-induced dynamics [25,30,31].

An additional and closely related challenge is *parameter identifiability* in multi-delay DDE models. In checkpoint systems, multiple combinations of reaction rates, delay values, and memory kernels can reproduce similar observable dynamics, such as mitotic arrest duration or APC/C activation profiles. This non-uniqueness is particularly pronounced for delays, which are often inferred indirectly and may compensate for one another. Addressing identifiability therefore requires mechanistic constraints, targeted perturbations, and integration of experimental priors to distinguish between competing delay structures and parameter sets.

Neural DDE surrogates and hybrid AI–mechanistic approaches (Section 8) offer promising directions but require closer integration with classical dynamical-systems theory.

### 9.5. Coupling DDEs with Spatial, Mechanical, and Population Scales

Integrating delayed checkpoint models with spatial, mechanical, and population dynamics remains a major open problem. Reaction–diffusion and mechanochemical models describe tension buildup, spatial recruitment, and spindle orientation [1,3,7,8,11,13], while population-level models describe how single-cell fates shape tissue growth or tumor progression.

Truly multi-scale frameworks must couple these layers consistently, determining which delays to retain, which to coarse-grain, and how feedback across scales modifies checkpoint timing [17]. Such models are critical for connecting single-cell checkpoint behavior with long-term phenotypic consequences.

### 9.6. Unified SAC–SPOC Models and Clinical Translation

A final challenge is constructing unified, predictive models of SAC, SPOC, and mitotic exit with clinical relevance. Many anti-mitotic treatments perturb delay-sensitive processes in these checkpoints [1,10,44,45]. Understanding how drugs alter delay-controlled feedback loops could explain why some treatments induce prolonged arrest and apoptosis, whereas others promote slippage and chromosomal instability. In addition, chromosome segregation errors arising during anaphase can feed forward to influence the timing of cytokinesis and mitotic exit, thereby linking SAC-controlled chromosome segregation to SPOC- and MEN-regulated late mitotic events [50].

Unified SAC–SPOC DDE models—constrained by mechanistic structure, reaction-network theory, and quantitative data—could serve as in silico platforms for probing how modulation of MCC turnover, APC/C recovery, or Tem1/MEN delays affects therapeutic outcomes [3,22,30,31]. Achieving this goal will require close collaboration across experimental, computational, and clinical communities. Key research directions for advancing delay-based SAC and SPOC models are outlined in Box 4.

Box 4 **Roadmap for Next-Generation Delay-Based Checkpoint Models.**

1. **Quantitative measurement of delays:** Mad2 activation, MCC transport, APC:MCC disassembly, Bfa1–Bub2–Tem1–MEN regulation.
2. **Multi-delay and multi-scale DDE frameworks:** Unified SAC–SPOC architectures capturing attachment, tension, and spindle orientation.
3. **Mechanochemical DDE models:** Explicit integration of force-dependent kinetics and viscoelasticity.
4. **AI-assisted inference:** Neural DDEs and Bayesian approaches for delay estimation, history reconstruction, and model selection.
5. **Stochastic delays:** Models incorporating intrinsic noise, spatial fluctuations, and resonance phenomena.
6. **Clinical applications:** Mapping delay perturbations to chromosomal instability and treatment response.
7. **Benchmarking and tools:** Standardized datasets, reproducible DDE toolboxes, and validated delay parameter sets.

A unified, mechanochemically grounded, delay-aware framework promises deeper insight into SAC and SPOC timing, robustness, and disease relevance.

## 10. Conclusions

Mitotic checkpoints, particularly the spindle assembly checkpoint (SAC) and the spindle position checkpoint (SPOC), safeguard chromosome segregation by coordinating kinetochore attachment, tension sensing, spindle orientation, and timely mitotic exit [1,3,10,44,45]. Experimental evidence demonstrates that these systems do not react instantaneously; instead, they integrate signals over characteristic timescales shaped by molecular conversion, spatial transport, mechanochemical coupling, and multi-step signaling cascades [4,5,6,7,8,10]. Classical ODE-based models have been instrumental in identifying the feedback motifs underlying ultrasensitivity, bistability, and checkpoint robustness [9,17,18,19,22], yet they encode temporal integration only implicitly.

Delay differential equations (DDEs) extend this framework by making biologically grounded time lags explicit. In SAC and SPOC, DDEs naturally represent delays in Mad2 activation, MCC assembly, APC/C inhibition and reactivation, tension buildup, spatial transport, and Bfa1–Bub2–Tem1 regulation [3,22,24,25,26,27]. Incorporating such delays enriches the dynamical repertoire of checkpoint models, revealing oscillations, prolonged transients, implicit checkpoint memory, variability in mitotic timing, and more complex behaviors that instantaneous models struggle to capture. When grounded in mechanochemical and spatial biology, delay parameters become interpretable and experimentally measurable quantities linked to specific biochemical or mechanical subprocesses.

At the same time, explicit delays introduce new challenges. Systems with multiple delays face parameter identifiability and calibration issues; the presence of noise motivates hybrid stochastic–DDE formulations; and mechanochemical and spatial coupling calls for multi-scale models that integrate molecular processes with micron-scale mechanics [17,24,25,26,27,30,31]. Numerical stiffness and high-dimensional history spaces further complicate simulation and bifurcation analysis. Addressing these challenges will require coordinated advances in experimental quantification of delay processes, mathematical theory, and computational methodology, including hybrid AI–DDE approaches for delay inference, surrogate modeling, and history reconstruction [3,17,22,30,31].

Looking forward, delay-aware and mechanochemically grounded SAC–SPOC models are poised to become central tools in quantitative cell biology. Integrating explicit time delays with high-content imaging, mechanical perturbations, and AI-assisted parameter inference will enable predictive models that connect molecular perturbations to cellular phenotypes and, ultimately, to tissue-level outcomes. In this perspective, delay differential equations are not merely refinements of existing models, but essential components of a temporally faithful and mechanistically complete understanding of mitotic checkpoint control and its implications for genomic stability. The core conceptual and methodological contributions of this work are summarized in Box 5.

Box 5 **Summary of Core Contributions.**

This review provides a unified perspective on how explicit time delays shape the function and dynamics of mitotic checkpoints. Key contributions include:

- **Biological grounding of delays:** Evidence synthesized from SAC and SPOC biology shows that checkpoint signaling integrates information over finite timescales arising from molecular conversion, mechanochemical coupling, spatial transport, and multi-step signaling.
- **DDE formulations for checkpoint dynamics:** Delay differential equations naturally capture history dependence in Mad2 activation, MCC assembly, APC/C inhibition, tension maturation, and SPOC–MEN regulation.
- **Dynamic consequences of delays:** Delay-induced phenomena such as oscillations, extended transients, checkpoint memory, variability in mitotic timing, and complex dynamics become accessible and interpretable in explicit DDE frameworks.
- **Integration with mechanochemical and AI methods:** Hybrid mechanochemical–DDE and AI-assisted approaches offer paths toward delay inference, model reduction, and multi-scale integration, providing a roadmap for next-generation predictive SAC–SPOC models.

## Data Availability

No new data were created or analyzed in this study. Data sharing is not applicable to this article.
